# Unequal Probability Marking Approach to Enhance Security of Traceback Scheme in Tree-Based WSNs

**DOI:** 10.3390/s17061418

**Published:** 2017-06-17

**Authors:** Changqin Huang, Ming Ma, Xiao Liu, Anfeng Liu, Zhengbang Zuo

**Affiliations:** 1 School of Information Technology in Education, South China Normal University, Guangzhou 510631, China; cqhuang@zju.edu.cn; 2 School of Information Science and Engineering, Central South University, Changsha 410083, China; minma@cs.stonybrook.edu (M.M); afengliu@mail.csu.edu.cn (A.L.); 3 Department of Computer Science, Stony Brook University, Stony Brook, NY 11794, USA; 4 The affiliated middle school, Hunan Normal University, Changsha 410012, China; zhengbangzuo@sina.com

**Keywords:** wireless sensor networks, unequal probability marking and migrating, network lifetime, storage, traceback time

## Abstract

Fog (from core to edge) computing is a newly emerging computing platform, which utilizes a large number of network devices at the edge of a network to provide ubiquitous computing, thus having great development potential. However, the issue of security poses an important challenge for fog computing. In particular, the Internet of Things (IoT) that constitutes the fog computing platform is crucial for preserving the security of a huge number of wireless sensors, which are vulnerable to attack. In this paper, a new unequal probability marking approach is proposed to enhance the security performance of logging and migration traceback (LM) schemes in tree-based wireless sensor networks (WSNs). The main contribution of this paper is to overcome the deficiency of the LM scheme that has a higher network lifetime and large storage space. In the unequal probability marking logging and migration (UPLM) scheme of this paper, different marking probabilities are adopted for different nodes according to their distances to the sink. A large marking probability is assigned to nodes in remote areas (areas at a long distance from the sink), while a small marking probability is applied to nodes in nearby area (areas at a short distance from the sink). This reduces the consumption of storage and energy in addition to enhancing the security performance, lifetime, and storage capacity. Marking information will be migrated to nodes at a longer distance from the sink for increasing the amount of stored marking information, thus enhancing the security performance in the process of migration. The experimental simulation shows that for general tree-based WSNs, the UPLM scheme proposed in this paper can store 1.12–1.28 times the amount of stored marking information that the equal probability marking approach achieves, and has 1.15–1.26 times the storage utilization efficiency compared with other schemes.

## 1. Introduction

With the development of the Internet of Things (IoT) [1,2], sensing devices have smaller volumes, stronger sensing abilities, larger sensing ranges and longer sensing times [3,4,5,6,7,8,9,10,11,12,13,14,15]. There is an enormous growth in the real-time or semi-real-time data obtained by networks, resulting in the centralized computing paradigm in clouding computing undergoing tremendous traffic pressure [16,17]. In this case, the fog computing mode is proposed to address the deficit of centralized computing for huge amounts of data [1,5,7]. In the fog computing mode, all levels of devices are distributed in the network, which reduces computation delay and network load [18,19,20]. Nowadays, many studies have been conducted on fog computing, with security issues being an important problem [21,22,23,24,25,26,27,28,29]. Thus, wireless sensor networks (WSNs) can be regarded as a special kind of fog computing, as the sensor node can be regarded as the edge devices and the base statin can be regarded as the information collection center. WSNs are widely used in a variety of applications, such as environmental monitoring, healthcare systems, military applications etc. [30,31,32,33]. Wireless sensor networks consist of a large number of sensor nodes that communicate with each other through multi-hop wireless links [34,35]. Due to the natural features of wireless sensor networks, nodes are often unattended and therefore become prone to various attacks [21,22,23,24,25,26,27,28,29,30,31], which includes clone attacks [23], select forwarding attacks [3], privacy preserving attacks [22,24,31], on-off attacks [36], black hole attacks, false data injection attacks [3,37], and so on.

The traceback approach is a promising method to hold many attacks back by monitoring the source of the malicious packet(s) or the attack path(s) [21,25,27,28,29,30]. The traceback approach can be divided into two categories: packet marking [21,30] and logging [29]. The main idea of packet marking is to record information (such as the node ID) of all visited nodes on packets [21,30]. The main idea of the logging is to store marked packets in relay nodes with a suitable data structure [29]. When being attacked, nodes can pinpoint the location of malicious nodes by inquiring upstream and broadcasting the information of malicious packet(s) in the traceback request to reconstruct the attack paths [21,30]. The packet marking method will be lengthened in the process of routing and increases the energy consumption of nodes near to the sink. The logging will lead to a phenomenon in which the logged marking information stored in nodes near to the sink are considerably greater than those in nodes far from the sink.

To overcome the above defects, Liu et al. proposed a logging and migration traceback (LM) scheme to improve the performance of the traceback scheme. The LM scheme [21] migrates the logged marking information to areas far from the sink, which saves more storage for storing marking information and utilizing the energy in remote areas more efficiently. The LM scheme can improve the security performance of the traceback scheme without reducing the network lifetime [21,35].

However, it is observed that much energy and storage is left in remote areas after the death of the whole network in the LM scheme. Therefore, the new strategy that can improve the utilization of storage and energy in remote areas is required to enhance the performance of the traceback approach.

In this paper, a new unequal probability marking logging and migration (UPLM) scheme is proposed. Compared with previous studies, the main contributions in this paper are as follows:

(1) Based on the logging and migration scheme, the UPLM scheme adopts an unequal probability marking approach. The new approach increases the marking probability of nodes far from the sink, thereby reducing the time needed to reconstruct attack paths when being attacked. Thus, this improves the security performance of the traceback scheme.

(2) According to theoretical analysis, the optimized unequal marking probability can improve the performance of previous schemes without reducing the network lifetime. The estimated network lifetime and the required storage capacity are given in this paper.

(3) The experimental simulation demonstrates that the UPLM scheme can improve the security performance, storage utilization, and network lifetime simultaneously. Compared with the LM traceback scheme, the energy utilization ratio of UPLM is above 95% and the amount of marking is increased by 2.1–5.2 times. Moreover, the network lifetime is longer than the LM scheme.

The rest of this paper is organized as follows: The system model and problem statement are given in Section 2. The unequal probability marking logging and migration (UPLM) scheme for tree-based wireless sensor networks is described in Section 3. Performance analysis is presented in Section 4. The paper is concluded in Section 5.

## 2. System Model and Problem Statement

### 2.1. System Model 

**Figure 1:**
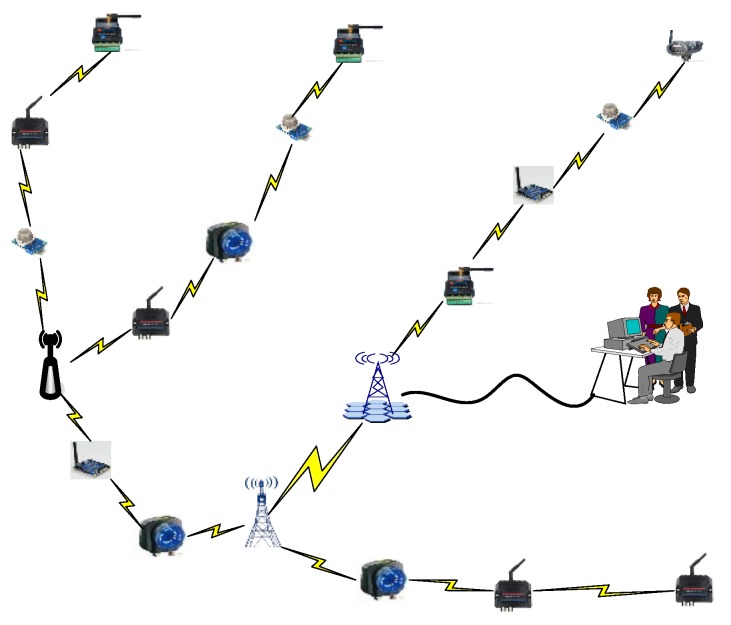
Tree-based wireless sensor networks.

(1) The network model studied in this paper is a tree-based wireless sensor network [34,38,39]. Tree-based wireless sensor networks consist of several linear networks. The root of the networks is the location named the sink (see Figure 1). One round is defined as the process where every node in this network generates a packet and forwards it to the sink [21,34]. The linear network is a special case of network design topology, which can be regarded as a type of basic research in the network. The results in the linear network can be applied in many scenarios, especially in the physical inspection of sensors and long distance multi-hop transmission. For example, many networks can be considered as linear networks, such as oil pipelines, the boundary line of a country, a road line, and underground coalmine tunnels. In comparison, the network routing can be considered as linear topology. This paper mainly studies long infrastructures, such as oil and gas pipelines, which have great significance. The studies in this paper are similar to a previous study [14]. In this study, the main research object was a network with small network traffic, which did not take into account the collision or interference from hidden nodes. We are prepared to consider these issues in the next step of research.

In real networks, the transmission of some pipelines is often not a single linear network. The nodes of the sensor near the base station can receive the information transmitted by a plurality of linear networks before transmitting the data to the base station. However, the tree network is such a network.

**Figure 2:**
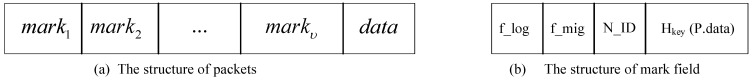
The structure of packets.

(2) The packet format is shown in Figure 2 [21]. Data and marking information are stored in different fields. Due to the resource-constrained nature of WSNs, a packet can be marked by no more than υ nodes. The structure of the marking field is shown in Figure 2b. In this figure, f_log is a flag indicating whether the marking information has been logged into a node or not. 0 means a node does not store this marking information, while 1 indicates that a node does store this marking information. The f_mig is a flag which denotes whether the marking information has been migrated by this node or not. 0 means the node does not migrate the marking information, while 1 means the node does migrate the marking information. N_ID is the ID of the node and H_key_ (P.data) denotes the hash code of the data field of the packet.

### 2.2. Energy Consumption Model and Related Definitions

A typical energy consumption model is adopted in this paper [21,34]. Equation (1) is the energy consumption model of sending data, while Equation (2) is the energy consumption model of receiving data.

(1) $$\left\{ \begin{array}{ll} E_{member}=lE_{elec}+l\varepsilon_{fs}d^{2} & if\;d<d_{0}\\ E_{member}=lE_{elec}+l\varepsilon_{amp}d^{4} & if\;d>d_{0} \end{array}\right.$$

(2) $$E_{R}(l)=lE_{elec}$$

where $E_{elec}$ represents the energy loss in transmitting circuits. If the transmitting distance is less than a threshold of $d_{0}$, the consumption of power amplification adopts the free space model. If the transmitting distance is more than a threshold of $d_{0}$, it adopts the multipath attenuation model. $\varepsilon_{fs}$ and $\varepsilon_{amp}$ are the energy required to amplify power in the two models, respectively. l denotes the number of bits of data. All parameters mentioned above refer to previous studies [21,34], which are shown in Table 1.

### 2.3. Problem Statement

The traceback approach mainly studies how to choose a traceback protocol in order to minimize the cost needed to determine the malicious source node after the victims being attacked. The goal of this paper can be categorized into several aspects:

(1) To prolong the network lifetime.

The network lifetime can be defined as the duration between the birth of the network and the moment of the death of the first node [21,34]. Assume $E_{i}$ is the energy consumption of node i and $E_{i\mathrm{nit}}$ is the energy consumption of node i. The goal of maximizing the network lifetime can be expressed by the following formula:

(3) max(T)=max0<i≤n(EinitEi)

(2) To minimize the requirement of storage capacity.

The storage capacity of every node in a homogeneous sensor network is the same. Furthermore, the storage capacity is subject to the node that consumes the most storage. Assuming that the storage capacity of node i is $s_{i}$, the second goal of this paper is to minimize the requirement of storage capacity S.

(4) $$\min(S)=\underset{0<i\leq n}{\min}(s_{i})$$

(3) To maximize logged marking information.

More logged marking information in the scheme will significantly reduce the time needed to reconstruct an attack path when being attacked and thereby will increase the security performance. The security problem is that when normal nodes send data packets to the sink, the sink can make full use of the received information to take measures to determine malicious nodes. However, there are some malicious nodes in the network, which can attack the normal nodes and then damage the data packet generated by normal nodes. When the damaged data packets are transmitted to the sink, the sink receives false information and takes false measures to deal with the event. Thus, false measures can negatively affect network events and can even be harmful to the entire network, resulting in the collapse of the entire network.

In order to delete malicious nodes, the proposed scheme in this paper increases the probability of nodes far from the sink and decreases the probability of nodes near to the sink. This results in the marking information of the data packet in the proposed scheme being more than that of the previous scheme. Thus, the required time for determining malicious nodes is less. For example, when one data packet is transmitted from the source node to the sink, the data packet can be marked by the information with the nodes’ IDs far from the sink with a higher probability, resulting in a greater amount of stored information in the nodes. Although the probability of nodes near to the sink will have declined, the proposed scheme always adopts the same probability as the previous scheme. Thus, the total stored information in the nodes along the routing path in the proposed scheme is much more than that of the previous scheme. When one malicious node attacks one normal node and sends a false data packet, the stored information in the nodes is greater. When the sink receives data packets, the sink can use enough information stored in the nodes to build a routing path quickly. This allows for the malicious node to be determined in a short time so that the network can take measures to delete the malicious nodes to ensure network security. Assuming $l_{i}$ is the marking information stored in node i, the goal of maximizing logged marking information is max(L) = $\underset{0<i\leq n}{\max}\left(\sum \left(l_{i}\right)\right)$.

In general, the optimization goal of the UPLM scheme can be summarized into the following formulas:

(5) {max(T)=max0<i≤n(EinitEi)min(S)=min0<i≤n(si)max(L)=max0<i≤n(∑(li))

## 3. UPLM Scheme

### 3.1. Research Motivation

**Figure 3:**
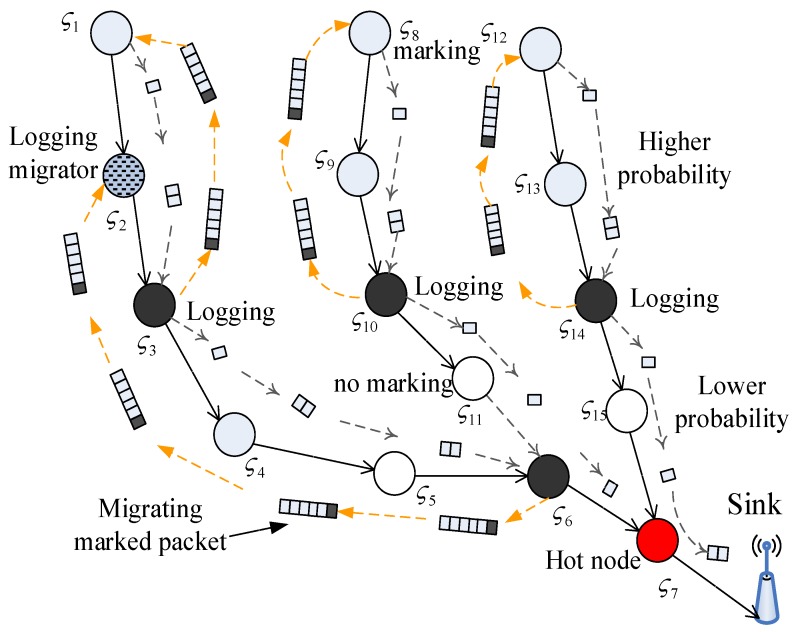
An example of the unequal probability marking logging and migration (UPLM) scheme.

Figure 3 illustrates the structure of tree-based WSNs. The equal marking probability approach in the LM scheme can lead to the following disadvantages, which become the primary motivation of the UPLM scheme:

(1) Although all nodes are given equal marking probability, the actual probability that a certain node is being marked is different. Assume the assigned probability that a node is being marked is $p_{lm}$ in the LM scheme. As illustrated in Figure 3, it is obvious that node $\varsigma_{6}$ is the convergence of two routing paths. Hence, all packets routing along these two paths will arrive at $\varsigma_{6}$ to be forwarded to the sink, while node $\varsigma_{6}$ will be marked by every packet at probability $p_{lm}$. The actual marking probability for node $\varsigma_{6}$ will be about two times higher than other nodes in these two paths. Similarly, node $\varsigma_{7}$ is about three times higher than other nodes. The probability of nodes marking data packets is different, which cause some nodes to not mark data packets when the sink receives data packets. Thus, the data packet must be transmitted several times in order for all the received nodes to contain all nodes’ IDs in the routing path. It can consume more energy and take a longer period of time.

(2) The storage in remote areas is not utilized effectively. The effects of the scheme that migrates the marking information to remote areas in the LM scheme is not satisfied, and much storage is wasted in remote areas. In the LM scheme, the probability of nodes is all the same when one data packet is transmitted from the source node to the sink. Due to the same probability of nodes, the marking information will be stored in nodes near to the sink. It will cause nodes far from the sink to be unable to store marking information. The storage of nodes far from the sink is wasted.

(3) There is still a large amount of energy remaining in remote areas after the death of the network. In the network, we can see that many packets are transmitted to the sink, which will cause nodes near to the sink to transmit too many packets. Thus, nodes near to the sink consume a considerable amount of energy. Nodes far from the sink consume less energy. When the energy of nodes near to the sink runs out, nodes far from the sink may have more energy.

To conquer the disadvantages of the LM scheme, an unequal probability marking logging and migration (UPLM) scheme for tree-based WSNs is proposed in this paper. In the UPLM scheme, a higher marking probability is given to nodes in remote areas, while a lower marking probability is applied to nodes in nearby areas. The former designation is based on the fact that nodes in remote areas have more remaining energy and storage. They can be utilized to transmit more packets and store more marking information by being given a higher marking probability. The latter designation is ascribed to the fact that nodes in nearby areas are much more likely to be the convergence of several routing paths. Hence, the actual marking probability of nodes in nearby areas will be higher than the assigned marking probability. A lower assigned marking probability will balance the actual marking probability in WSNs, thereby reducing energy consumption and storage requirement in nearby areas significantly.

Compared to the LM scheme, the probability of nodes are the same. Figure 3 shows the structure of UPLM. It can be seen that the probability of nodes far from the sink is higher, while the probability of nodes near to the sink is lower. Thus, we can see that in Figure 3, $\varsigma_{1}$, $\varsigma_{2}$, $\varsigma_{8}$, $\varsigma_{9}$, $\varsigma_{12}$, and $\varsigma_{13}$ have a higher probability, with the probability of those nodes being 1 in the UPLM scheme. Thus, we can see that the data packet can be marked with the information with those nodes. However, when data packets are transmitted to nodes near to the sink, those nodes have lower probability, meaning that nodes near to the sink do not mark data packets when they receive them. Thus, the nodes $\varsigma_{5}$, $\varsigma_{11}$, and $\varsigma_{15}$ do not mark data packets.

The effectiveness of the UPLM scheme can be demonstrated with the tree-based WSNs in Figure 3. There are three linear branches in Figure 3. The first branch is the path: $\varsigma_{1}$ → $\varsigma_{2}$ → $\varsigma_{3}$ → $\varsigma_{4}$ → $\varsigma_{5}$ → $\varsigma_{6}$ → $\varsigma_{7}$ → sink; the second one is the path: $\varsigma_{8}$ → $\varsigma_{9}$ → $\varsigma_{10}$ → $\varsigma_{11}$ → $\varsigma_{6}$; and the third one is the path: $\varsigma_{12}$ → $\varsigma_{13}$ → $\varsigma_{14}$ → $\varsigma_{15}$ → $\varsigma_{7}$. According to the unequal marking probability approach in the UPLM scheme, the nodes $\varsigma_{1}$, $\varsigma_{2}$, $\varsigma_{3}$, $\varsigma_{8}$, $\varsigma_{9}$, $\varsigma_{10}$, $\varsigma_{12}$, $\varsigma_{13}$, and $\varsigma_{14}$ are assigned with a marking probability of 1 (in other word, packets arriving at these nodes will be marked) and other nodes will be assigned with lower marking probability. Both the lengths of the marked fields of the packet travelling from $\varsigma_{1}$ to $\varsigma_{3}$ via $\varsigma_{2}$ and the packets travelling from $\varsigma_{8}$ to $\varsigma_{10}$ via $\varsigma_{9}$ are 3. Similarly, the length of the marked fields of the packet travelling from $\varsigma_{12}$ to $\varsigma_{14}$ is 3. 

If we set υ = 3, the logging will be processed when the length of the marked field reaches 3 and the marking information will be stored in nodes $\varsigma_{3}$, $\varsigma_{10}$, and $\varsigma_{14}$. Compared with the LM scheme, more marking information will be logged. The same marking probability of the peripheral area in the UPLM scheme is adopted with the LM scheme. As a result, the UPLM scheme can increase the number of logged marking information and therefore enhances the network performance, such as extending the network lifetime.

### 3.2. The Pseudocode of the UPLM Scheme

Based on the arguments above, the UPLM scheme will be presented in this subsection. In the UPLM scheme, when receiving packets, different probabilities will be adopted in different nodes according to their distances from the nodes to the sink. Once the number of marking information stored in a node exceeds υ, a part of them will be migrated to the node whose distance to sink is w hops larger than the original node. Algorithm 1 gives the pseudocode of the UPLM scheme.

**Algorithm 1.** Unequal probability marking logging and migration (UPLM) scheme.1:**For** each packet $P_{i}$ of node $\varsigma_{i}$ received2:  node $\varsigma_{i}$ computes marking probability $f_{i}^{\alpha }$ using Equation (6);3:  marks packet $P_{i}$ with probability $f_{i}^{\alpha }$;4:  **If** the number of markings storage in node $\varsigma_{i}$
≥
υ;5:   node $\varsigma_{i}$ marks packet $P_{i}$ with his probability;6:    k = w;7:   **While**
k > 0 **Do**8:    The marking information of packet $P_{i}$ is forwarded backward with 1-hop;9:    k = k − 1;10:   **End** while11:  **End** if12:  node $\varsigma_{i}$ sends packet $P_{i}$ to next node;13:**End** for

### 3.3. The Probability Analysis of the UPLM Scheme

In this subsection, the computation method of marking probability in the UPLM scheme will be described. In the UPLM scheme, the factors that influence the marking probability are as follows: 

(1) The 1-hop nodes (n-hop nodes are defined as the nodes that can transmit packets to the sink in n-hop) will transmit far more packets than other nodes. Hence, the marking probability of them should not be larger. Similarly, the marking probability of 2-hop nodes should also not be large. Migration is needed to further reduce the pressure of hotspots. To achieve the goal of the UPLM scheme, nodes in remote areas should be assigned with a higher marking probability.

(2) As the packets of every upstream node will be marked at a probability of $\tau_{i}$ by node i, the total marking probability of node $\varsigma_{i}$ is still high, even if the assigned marking probability is low. Hence, the strategy is to increase the assigned marking probability in remote areas and decrease the marking probability in areas near to the sink in the UPLM scheme. According to the reasoning above, assume the distance of a node with $\hbar_{i}$-hop from the sink is $\varsigma_{i}$. If the marking probability adopted by the LM scheme is ∂, w is the migrated hop of marking information and ν is the hop counts that marking information is logged in nodes. The marking probability of $\varsigma_{i}$ in the UPLM scheme is:

(6) $$f_{i}^{\alpha }=\{\begin{array}{cc} \frac{\partial }{w} & if\;\hbar_{i}\leq w\\ \frac{\hbar_{i}\partial }{3v} & if\;w<\hbar_{i}\leq w+3\nu \\ 1 & if\;\hbar_{i}>w+3\nu \end{array}$$

The marking probability of nodes in the UPLM scheme is divided into three aspects. The first one includes nodes in the range of ≤w hops areas near to the sink. This section should reduce the probability to increase network lifetime. Thus, we know the probability of nodes in the LM scheme is ∂, if there are w hops in this area. The total marking probability in the LM scheme is ∂w. However, in this area, we used $\frac{\partial }{w}$ in the UPLM scheme to reduce marking information in this area, to increase the network lifetime. If nodes in the area near to the sink have $\hbar_{i}>w+3\nu$ hops, nodes are far from the sink when one packet passes by the nodes in the range of $\hbar_{i}>w+3\nu$ hops. Nodes mark the packets with a probability of 1, making it feasible. It does not affect the network lifetime. If nodes in the area near to the sink have $w<\hbar_{i}\leq w+3\nu$ hops, the total marking probability of nodes in the LM scheme is $\hbar_{i}\partial$. There are 3v hops in this area. In the UPLM scheme, the probability of nodes in this area is $\frac{\hbar_{i}\partial }{3v}$. The total marking information in this area in the UPLM scheme is equal to the total marking information in this area in the LM scheme; it does not affect the network lifetime and storage space.

### 3.4. The Improved UPLM Scheme

Although the UPLM scheme can balance storage utilization to some extent, there is still an unbalanced situation as a greater amount of storage is required by the nodes near the sink, which seriously increases the cost of the whole system. Therefore, an improved UPLM scheme is proposed to solve this issue. In the former UPLM scheme, the process of migration starts when the number of marked packets exceeds a given threshold, which causes a phenomenon in which nodes near the sink more frequently run the process. This strategy significantly increases the storage and energy consumption of those nodes. We propose an improved UPLM scheme, with a buffer area introduced into this scheme. A buffer area is a set of nodes. Once packets are forwarded into the buffer area, the marking information of these packets are entirely stored or migrated. We no longer control the process by setting a threshold. In this paper, nodes that have a distance to the sink within 5 hops are set to be the buffer area. The buffer area can assist and balance the process of storage and transference, which further improves the network performance [40,41,42,43].

## 4. Performance Analysis

### 4.1. Analysis of the Amount of Marking Information Processed by Nodes

In this subsection, the number of marking information processed by nodes, logged marking information, and migrated marking information of node i in a round will be analyzed.

**Theorem** **1.** *In a single-branched network, the number of packets node i sent and received are $Q_{i}^{r}$ and $Q_{i}^{s}$, respectively. The number of marking information sent and received by node i are ${ℑ}_{i}^{r}$ and ${ℑ}_{i}^{s}$, respectively. The storage required is ${ℜ}_{i}$. The number of migrated marking information is ${ℑ}_{i}^{mig}$. The relationships of these variables are presented by the following formulas*:

(7) $$\left\{ \begin{array}{l} Q_{i}^{r}=i-1\\ Q_{i}^{s}=i \end{array}\right.$$

(8) $${ℑ}_{i}^{r}=\left\{ \begin{array}{l} (n-1)\tau_{n-1}\;if\;i=n\\ \sum_{k=1}^{i-1}((\sum_{z=k}^{i-1}\tau_{z})\mathrm{mod}\;\upsilon )\;else \end{array}\right.$$

(9) $${ℑ}_{i}^{s}=\left\{ \begin{array}{l} (n-1)\tau_{n-1}+n\tau_{n}\;if\;i=n\\ (n-1)\tau_{n-1}\;if\;i=n-1\\ \sum_{k=1}^{i-1}\left((\sum_{z=k}^{i-1}\tau_{z})\mathrm{mod}\;\upsilon +\tau_{i}\right)\;\mathrm{else} \end{array}\right.$$

(10) $${ℜ}_{i}=\left\{ \begin{array}{l} {ℑ}_{n-1}^{r}\;if\;i=n-1-w\\ \sum_{k=1}^{i+w}b\;\mathrm{else} \end{array}\right.|if\;\left((\sum_{z=k}^{i+w-1}\tau_{z})\;/\;\upsilon \;\leq \;(\sum_{z=k}^{i+w}\tau_{z})\;/\;\upsilon \;\right)\;then\;b=\upsilon \;else\;b=0$$

(11) $${ℑ}_{i}^{mig}=\sum_{k=i-w+1}^{i-1}{ℜ}_{k}$$

**Proof.**  Single-branched network contains n nodes and one sink. The packets received by node i are from other i−1 nodes. Thus, node i receives i−1 packets in a round. When node i receives or generates a packet, it will forward it to the next node using shortest routing algorithm [21]. As a result, the number of packets that node i forwards is i.

In a single-branched network, packets will be marked at a certain probability. Assume the probability that node i marks packets is $\tau_{i}$. In the process, when a packet generated by node 1 is forwarded to node i, the total marking information is as follows:

(12) $$\sum_{z=1}^{i-1}\tau_{z}=\tau_{1}+\tau_{2}+\tau_{3}+\tau_{4}+...+\tau_{i-1}$$

The logged marking information will be migrated backwards with w-hop once the number of marking information exceeds υ. After completing forwarding packets generated by node 1 to node i, the marking information received by node i is $\sum_{z=1}^{i-1}\tau_{z}\mathrm{mod}\;\upsilon$.

In the process where a packet generated by node 2 is forwarded to node i, the total marking information is $\tau_{1}+\tau_{2}+\tau_{3}+\tau_{4}+...+\tau_{i-1}$ = $\sum_{z=2}^{i-1}\tau_{z}$. Similarly, the marking information from node 2 received by node i is $\sum_{z=2}^{i-1}\tau_{z}\mathrm{mod}\;\upsilon$. The marking information from node 3 received by node i is $\sum_{z=3}^{i-1}\tau_{z}\mathrm{mod}\;\upsilon$. In the process where the packet generated by node i−1 is forwarded to node i, the marking information received by node i is $\tau_{i-1}\;\mathrm{mod}\;\upsilon$. The total marking information received by node i is ${ℑ}_{i}^{r}=\sum_{k=1}^{i-1}\left((\sum_{z=k}^{i-1}\tau_{z})\mathrm{mod}\;\upsilon \right)$.

As packets will travel through node n−1 when being forwarded to node n, all the marking information in the packets will be migrated to nodes far away from the sink and only the data field will be sent to node n. All marking information will be logged once the packets arrive at node n−1. The marking probability of node n−1 is $\tau_{n-1}$. The number of packets received by the node is n−1. Hence, the number of marking information received by node n is $(n-1)\tau_{n-1}$.

Likewise, migration will be processed once the number of marking information reaches υ. For node i, the number of marking information is $T=\sum_{k=1}^{i-1}\left((\sum_{z=k}^{i-1}\tau_{z})\mathrm{mod}\;\upsilon \right)$. When k = 0, all marking information that arrives at the node will be logged and migrated. Therefore, the number of marking information sent by node i is $\tau_{i}$. If T<υ, $T+\tau_{i}$ marking information will be forwarded.

According to the reasoning above, the amount of marking information received by node i is ${ℑ}_{i}^{r}=\sum_{k=1}^{i-1}\left((\sum_{z=k}^{i-1}\tau_{z})\mathrm{mod}\;\upsilon \right)$ and hence, the amount of marking information sent by node i is ${ℑ}_{i}^{s}=\sum_{k=1}^{i-1}\left((\sum_{z=k}^{i-1}\tau_{z})\mathrm{mod}\;\upsilon +\tau_{i}\right)$.

All the marking information in the packet will be migrated to nodes with larger hops to the sink when the packet arrives at node n−1. The packet will be marked at $\tau_{n-1}$ probability, before being forwarded to the next node n. The number of marking information sent by node n−1 is $(n-1)\tau_{n-1}$.

The packet will be marked at probability $\tau_{n}$ after being received by node n, before being forwarded to other nodes. As the number of marking information received by node n is $(n-1)\tau_{n-1}$ and all data in these packets will be marked at probability $\tau_{n}$, the total amount of marking information is $\left(n-1)(\tau_{n-1}+\tau_{n}\right)$. In addition, node n will generate its own packets and forward them out at a probability of $\tau_{n}$. Therefore, the amount of marking information forwarded bynode n is ${ℑ}_{i}^{s}=(n-1)\tau_{n-1}+n\tau_{n}$.

The analysis of the migration of node i is described in this paragraph. As the marking information will be migrated backwards with w-hop when the number of marking information reaches υ, the number of migrated marking information of node i is the summation of the amount of migrated marking information of node i−w+1, i−w+2, …, i−1. The total amount of migrated marking information of these nodes is ${ℜ}_{i-w+1}$, ${ℜ}_{i-w+2}$, …, ${ℜ}_{i-1}$. Therefore, the total amount of migrated marking information of node i is ${ℑ}_{i}^{mig}=\sum_{k=i-w+1}^{i-1}{ℜ}_{k}$.

The analysis of storage is described in this paragraph. As node i will store the marking information from node i+w, migration will be conducted when the amount of marking information reaches υ after node i+w receives the packet. The number of marking information migrated to node i is υ and no marking information is migrated from other nodes. The following formula describes the idea:

$${ℜ}_{i}=\sum_{k=1}^{i+w}b\;|\;if\;\left((\sum_{z=k}^{i+w-1}\tau_{z})\;/\;\upsilon \;\leq \;(\sum_{z=k}^{i+w}\tau_{z})\;/\;\upsilon \right)\;then\;b=\upsilon \;else\;b=0$$

Hence, the marking information stored at node n−1−w is entirely from node n−1, which is the amount of migrated marking information of node n−1. If i=n−1−w, the storage requirement of node n−1−w is ${ℜ}_{i}={ℑ}_{n-1}^{r}$.■

**Theorem** **2.** *In the UPLM scheme, the number of packets received and sent by node i is ${Μ}_{i}^{r}$ and ${Μ}_{i}^{s}$ in the tree network, respectively. The amount of marking information received and sent by node i is ${ℵ}_{i}^{r}$ and ${ℵ}_{i}^{s}$ in the tree network, respectively. The storage required is $\Xi_{i}$ in the tree network. The number of migrated marking information is $\Omega_{i}$ in the tree network. In the following formulas, $\Psi_{i}$ represents the set of nodes whose packets are forwarded to the sink through node i. $\left|\Psi_{i}\right|$ represents the number of elements in the set $\Psi_{i}$; $\eta_{i}$ denotes the set of paths that pass through node i; $\left|\eta_{i}\right|$ represents the number of elements in the set $\eta_{i}$; and $\tau_{i}$ represents the probability that node i will mark the packet.*

(13) $$\left\{ \begin{array}{l} {Μ}_{i}^{r}=|\Psi_{i}|\\ {Μ}_{i}^{s}=|\Psi_{i}|+|\eta_{i}| \end{array}\right.$$

(14) $$\{\begin{array}{c} {ℵ}_{i}^{r}=\sum_{l}{ℑ}_{i,l}^{r}(l\in \eta_{i})\\ {ℵ}_{i}^{s}=\sum_{l}{ℑ}_{i,l}^{s}(l\in \eta_{i}) \end{array}$$

(15) $$\Omega_{i}=\sum_{l}{ℑ}_{i,l}^{mig}(l\in \eta_{i})$$

(16) $$\Xi_{i}=\sum_{l}{ℜ}_{i,l}(l\in \eta_{i})$$

**Proof.**  In the UPLM scheme, the number of packets received by node i is the number of nodes whose packets will be forwarded to the sink through node i, namely $\left|\Psi_{i}\right|$. Hence, ${Μ}_{i}^{r}$ = $\left|\Psi_{i}\right|$. The number of packets sent by node i is ${Μ}_{i}^{s}$ = $\left|\Psi_{i}|+|\eta_{i}\right|$ ($\left|\eta_{i}\right|$ is the number of packets sent by node i because node i will forward a packet to every path).

There are $\left|\eta_{i}\right|$ paths going through node i. For a certain path l, the amount of marking information received by node i is ${ℑ}_{i,l}^{r}$. The number of marking information received by node i is the summation of all paths, namely $\sum_{l}{ℑ}_{i,l}^{r}(l\in \eta_{i})$. Similarly, the number of marking information sent by node i is $\sum_{l}{ℑ}_{i,l}^{S}(l\in \eta_{i})$.

As the amount of migrated marking information in path l that passes through node i is ${ℑ}_{i,l}^{mig}$, and there are $\left|\eta_{i}\right|$ paths going through node i, therefore, its amount of migrated marking information is $\sum_{l}{ℑ}_{i,l}^{mig}(l\in \eta_{i})$. Similarly, as the amount of stored marking information in path l that passes through node i is ${ℑ}_{i,l}^{mig}$ and there are $\left|\eta_{i}\right|$ paths going through node i, its amount of stored marking information is $\sum_{l}{ℑ}_{i,l}^{mig}(l\in \eta_{i})$.

The storage required is $\Xi_{i}$, the storage space of node i in path l is ${ℜ}_{i,l}$ and there are $\left|\eta_{i}\right|$ paths going through node i. Therefore, its storage space is $\sum_{l}{ℜ}_{i,l}(l\in \eta_{i})$. ■

### 4.2. Energy Consumption and Network Lifetime

**Theorem** **3.** *In the UPLM scheme, the initial energy consumption of node i is $E_{init}$, the energy consumption of node i is $e_{i}$ and the network lifetime of node i is $\ell_{i}$. Furthermore, α and β are the number of data packets and marking information, respectively. They are as follows*:

(17) $$e_{i}=\{\begin{array}{c} (\alpha {Μ}_{i}^{r}+\beta {ℵ}_{i}^{r}+\beta \Omega_{i})E_{elec}+(\alpha {Μ}_{i}^{r}+\beta {ℵ}_{i}^{r}+\beta \Omega_{i})\varepsilon_{fs}d^{2}+(\alpha {Μ}_{i}^{s}+\beta {ℵ}_{i}^{s}+\beta \Omega_{i})E_{elec}\;d<d_{0}\\ (\alpha {Μ}_{i}^{r}+\beta {ℵ}_{i}^{r}+\beta \Omega_{i})E_{elec}+(\alpha {Μ}_{i}^{r}+\beta {ℵ}_{i}^{r}+\beta \Omega_{i})\varepsilon_{amp}d^{4}+(\alpha {Μ}_{i}^{s}+\beta {ℵ}_{i}^{s}+\beta \Omega_{i})E_{elec}\;d>d_{0} \end{array}$$

(18) $$\ell_{i}=\frac{E_{init}}{e_{i}}$$

**Proof.**  In the UPLM scheme, according to Equations (1) and (2), the energy consumption of node i is the sum of the energy consumption for receiving data and the energy consumption for sending data. Thus, the energy consumption of node i is as follows:

(19) $$e_{i}=E_{member}+E_{R}$$

According to Equations (1) and (2),

(20) $$e_{i}=\{\begin{array}{ll} lE_{elec}+l\varepsilon_{fs}d^{2}+lE_{elec} & d<d_{0}\\ lE_{elec}+l\varepsilon_{amp}d^{4}+lE_{elec} & d>d_{0} \end{array}$$

According to Theorem 2, the number of packets received and sent by a node i is ${Μ}_{i}^{r}$ and ${Μ}_{i}^{s}$ in the tree network, respectively. The amount of marking information received and sent by a node i is ${ℵ}_{i}^{r}$ and ${ℵ}_{i}^{s}$ in the tree network, respectively. The storage required is $\Xi_{i}$ in the tree network. The number of migrated marking information is $\Omega_{i}$ in the tree network.

(21) $$e_{i}=\{\begin{array}{cc} (\alpha {Μ}_{i}^{r}+\beta {ℵ}_{i}^{r}+\beta \Omega_{i})E_{elec}+(\alpha {Μ}_{i}^{r}+\beta {ℵ}_{i}^{r}+\beta \Omega_{i})\varepsilon_{fs}d^{2}+(\alpha {Μ}_{i}^{s}+\beta {ℵ}_{i}^{s}+\beta \Omega_{i})E_{elec} & d<d_{0}\\ (\alpha {Μ}_{i}^{r}+\beta {ℵ}_{i}^{r}+\beta \Omega_{i})E_{elec}+(\alpha {Μ}_{i}^{r}+\beta {ℵ}_{i}^{r}+\beta \Omega_{i})\varepsilon_{amp}d^{4}+(\alpha {Μ}_{i}^{s}+\beta {ℵ}_{i}^{s}+\beta \Omega_{i})E_{elec} & d>d_{0} \end{array}$$

Due to the initial energy consumption of node i being $E_{init}$, the network lifetime of a node i is as follows:

(22) $$\ell_{i}=\frac{E_{init}}{e_{i}}$$

## 5. Experiment Results

Omnet++ is adopted to evaluate the UPLM scheme performance [44]. In this simulation, if not specified, the experimental simulation scenario is as follows: 600 nodes are deployed in a network, each node produces a data packet in a data collection cycle and these packets are sent to the sink using multiple hops. The transmission radius of a node is r = 50 m, v = 3, w = 2. The maximum storage space is 300 bits. However, the probability of nodes is 0.5 in the LM schemes. 

**Figure 4:**
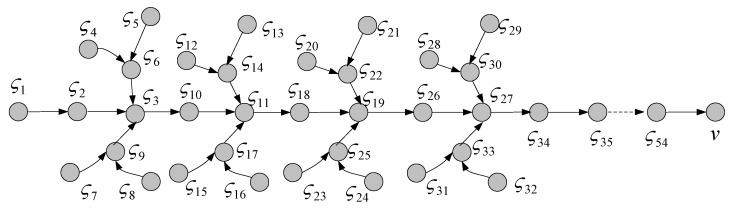
Tree-based wireless sensor network.

In this section, the performance of the UPLM scheme was analyzed by comparison with previous schemes. Two schemes are used in comparison. The first scheme is the baseline version probability traceback (BVP) scheme. This involves a data packet being marked with relayed nodes’ ID information in the routing path with some probability when one data packet is transmitted to the sink. When the marking information of one data packet reaches v, the marking information will be stored in this node. Following this, the data packet will be transmitted to the next node. However, the marking information in the BVP scheme cannot be migrated to nodes far from the sink [29]. The second scheme is the LM scheme. This scheme is based on tree-based WSNs (as illustrated in Figure 4) and proposed by a previous study [21]. The LM scheme is also referred to as the equal probability scheme in this paper. In this scheme, nodes mark packets with equal probability and require the w-hop migration, which will migrate the marking information to other nodes when the number of marking information reaches v.

### 5.1. Analysis of the Amount of Marking Information Processed by Nodes

**Figure 5:**
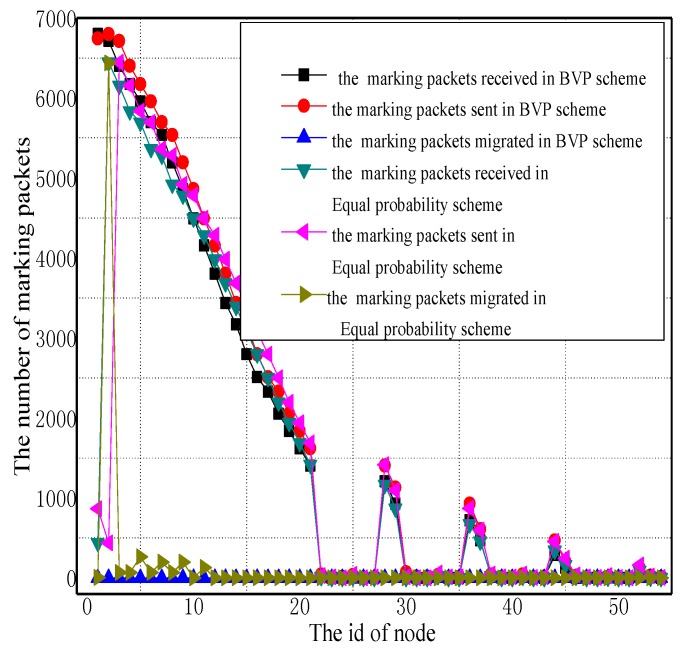
The number of marking packets received and sent in different schemes.

**Figure 6:**
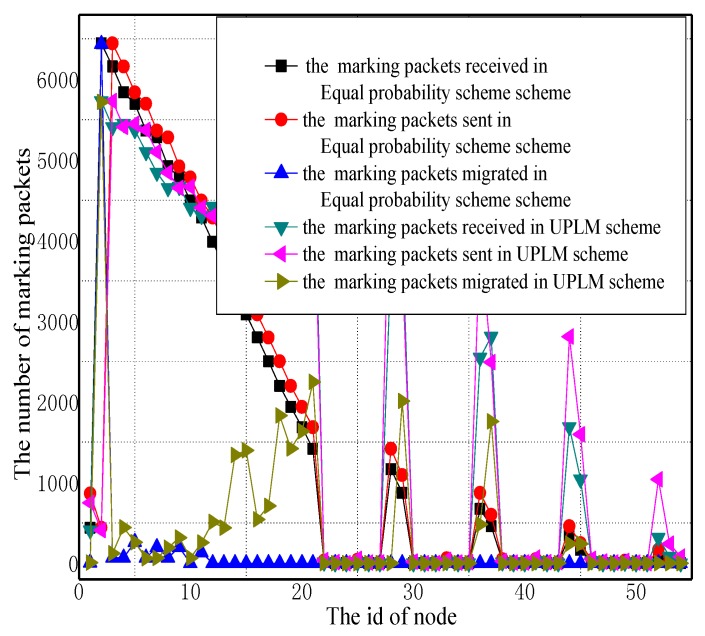
The number of marking packets received and sent in different schemes.

Figure 5 and Figure 6 illustrate the total amount of marking information received by nodes in all three schemes. It can be inferred from these figures that the amount of marking information received and sent by the nodes near to the sink is at the maximum. It is worthy to notice that the nodes in the UPLM scheme that have a longer distance to the sink receive a larger amount of marking information when compared with other schemes. This can be attributed to the following fact. On the one hand, lower marking probabilities were assigned to nodes closer to the sink, while higher marking probabilities were assigned to nodes farther away from the sink. On the other hand, the marking information stored in nodes will be migrated once its length exceeds υ. Therefore, the amount of marking information stored in remote areas will increase. In the UPLM scheme, the logged marking information were distributed more evenly in whole WSNs. In addition, the maximum storage required to store the marking information is no greater than other schemes.

**Figure 7:**
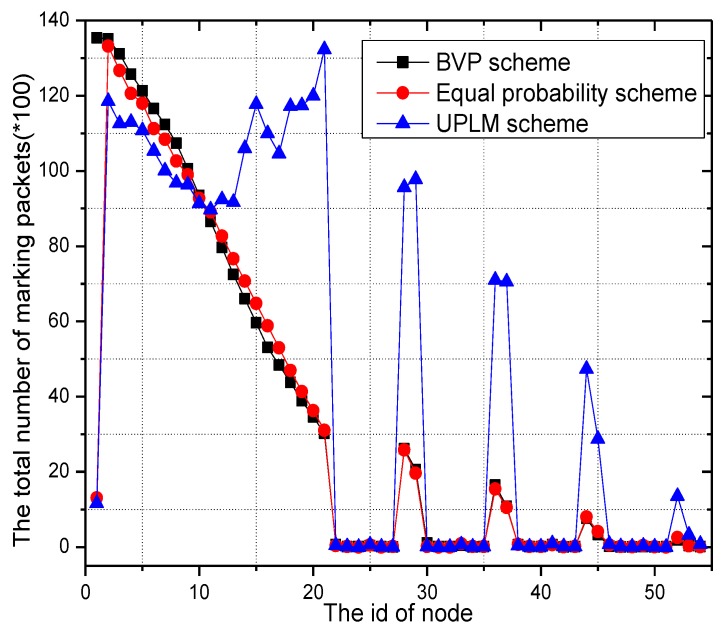
The total number of marking packets received and sent in different schemes.

Figure 7 illustrates the statistical distribution of receiving packets, sending packets, and migration marking information in different areas of the network. It can be observed from the figure that in the UPLM scheme, the amount of marking information that every node processed is balanced.

**Figure 8:**
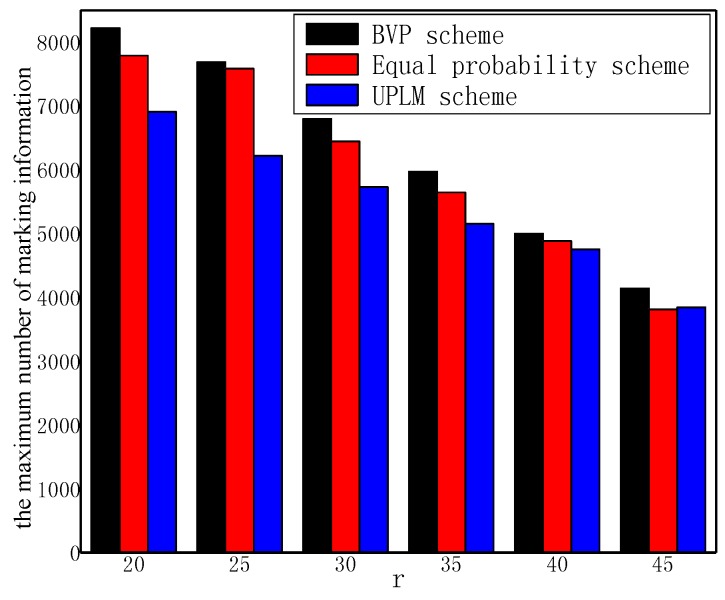
The maximum number of marking information under different transmission radii.

If we sort the amount of marking information processed by each node in descending order, the largest one is called the maximum marking information (MMI). Figure 8 illustrates the maximum amount of marking information in the network in several traceback schemes. These facts can be inferred from Figure 8. The maximum amount of marking information in the BVP scheme is the greatest, while the UPLM scheme achieves the lowest value. 

**Figure 9:**
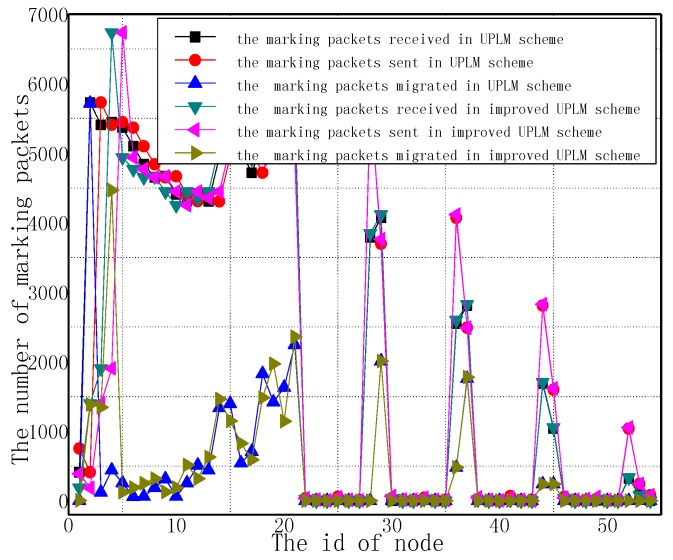
The number of marking information received and sent by nodes.

Figure 9 illustrates the total marking information received by nodes in the improved UPLM scheme and the UPLM scheme. It is obvious that the amount of marking information in both schemes are nearly equal. Due to the buffer area in the improved UPLM scheme, marking information will be migrated to nodes far away from the sink. The process of migration will utilize the remaining energy of these nodes effectively, and thereby optimizes the network lifetime and shows the effectiveness of the improved UPLM scheme.

**Figure 10:**
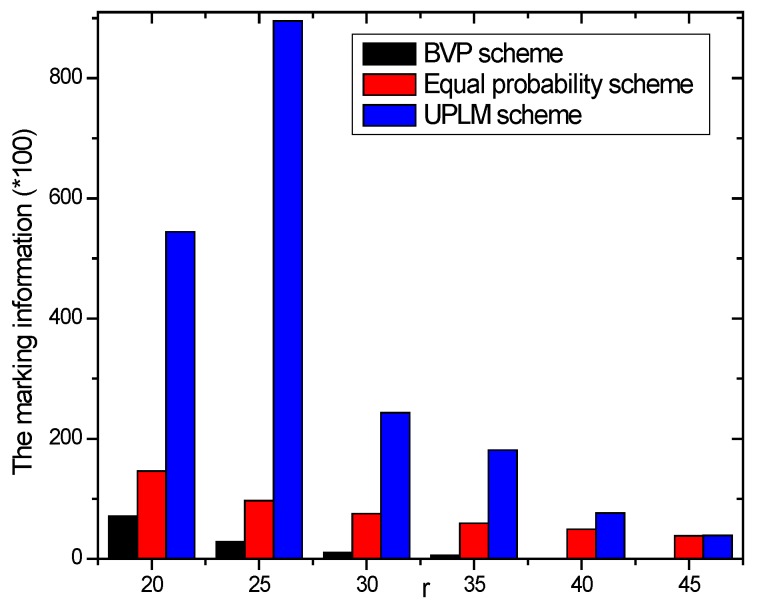
The total marking information stored under different transmission radii.

Figure 10 illustrates the total marking information stored in the network under different transmission radii. It is obvious that the total amount of stored marking information of the UPLM scheme is 1.00943–9.22717 times higher than that of the equal marking probability approach, which indicates the UPLM scheme can utilize the remaining storage of non-hotspot areas more effectively to store more marking information. Thus, the convergence time needed to determine the malicious node is reduced when a certain node is being attacked.

**Figure 11:**
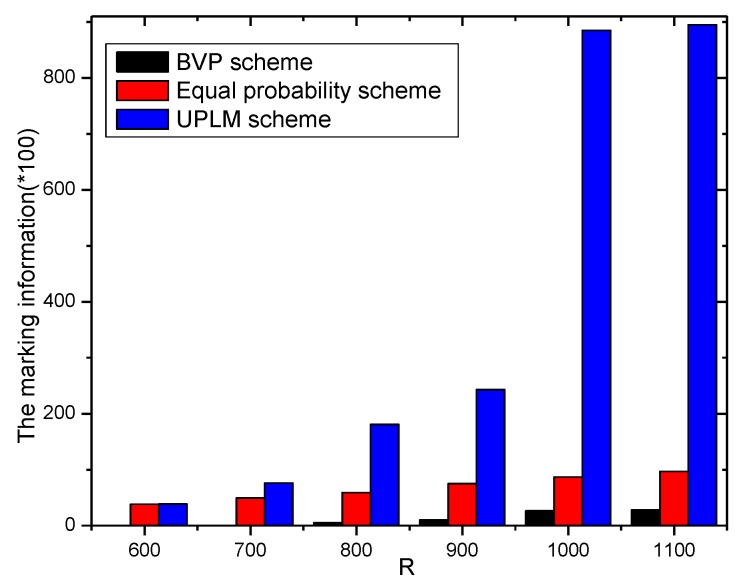
The stored total marking information under different network radii.

**Figure 12:**
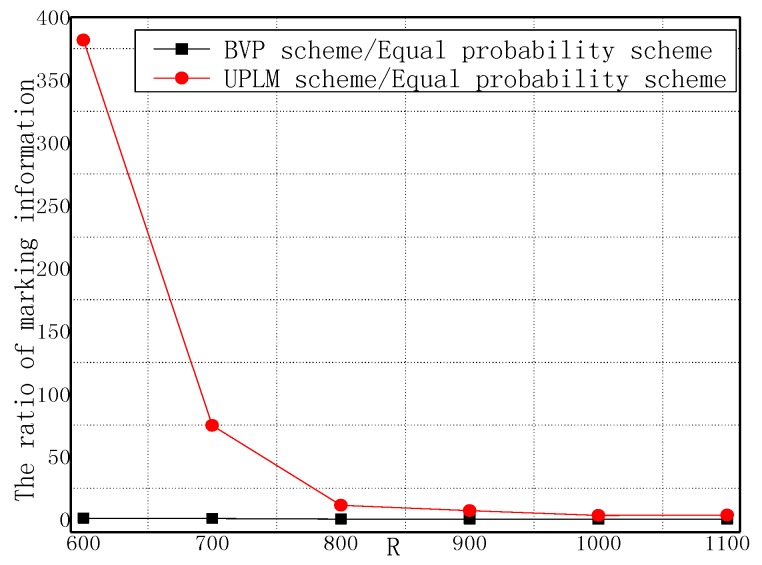
The ratio of total stored marking information in the UPLM scheme and the improved UPLM scheme.

Figure 11 and Figure 12 illustrate the comparison of the total marking information in the UPLM scheme and the equal marking probability approach under different network radii. It can be inferred that the total marking information in the UPLM scheme under different network radii is 3.4186–381.6 times more than that of the equal marking probability approach.

### 5.2. Energy Consumption and Network Lifetime

**Figure 13:**
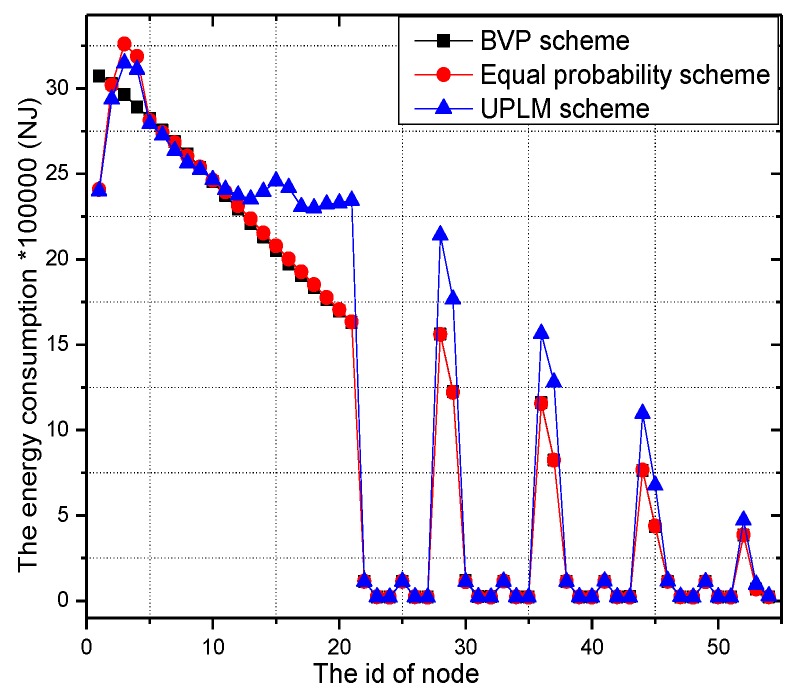
The total energy consumption of nodes in different schemes.

Figure 13 illustrates the energy consumption in different areas and different approaches. As the energy consumption of data collection in different traceback approaches are equal, this part is only described once in the section. Figure 13 shows the maximum energy consumption in different schemes. The UPLM scheme and BVP scheme are approximately equal. Although the energy consumption is nearly equal in these three schemes, the energy consumption patterns in the BVP scheme and equal marking probability approach are significantly different in different areas, which implies an imbalance of energy consumption in these two schemes. A small amount of energy is consumed in remote areas and a large amount of energy is consumed in nearby areas. However, the energy consumption pattern of the UPLM scheme is balanced, which infers that it is capable of balancing energy consumption.

**Figure 14:**
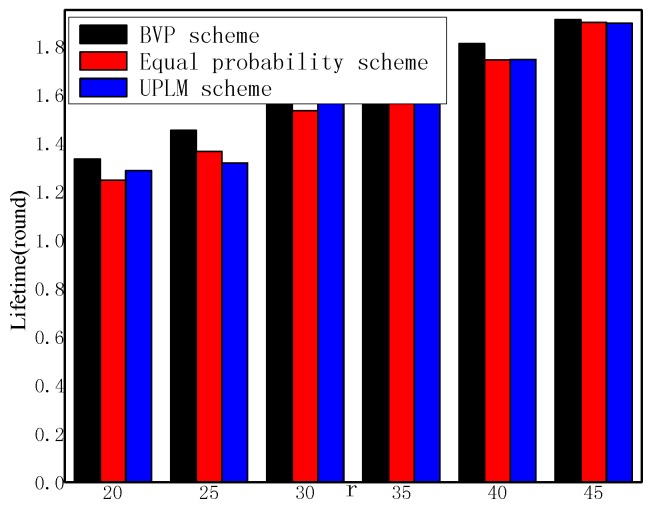
The network lifetime under different transmission radii.

**Figure 15:**
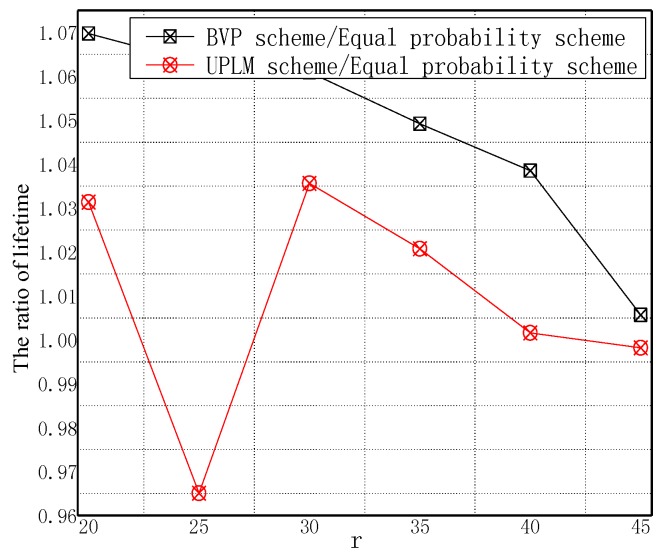
The ratio of network lifetime under different transmission radii.

Figure 14 and Figure 15 illustrate the network lifetime under different transmission radii r in different schemes. The network lifetime of the UPLM scheme, BVP scheme, and equal marking probability approach under different transmission radii are only slightly different from each other, which implies that the UPLM scheme will not affect the network lifetime.

**Figure 16:**
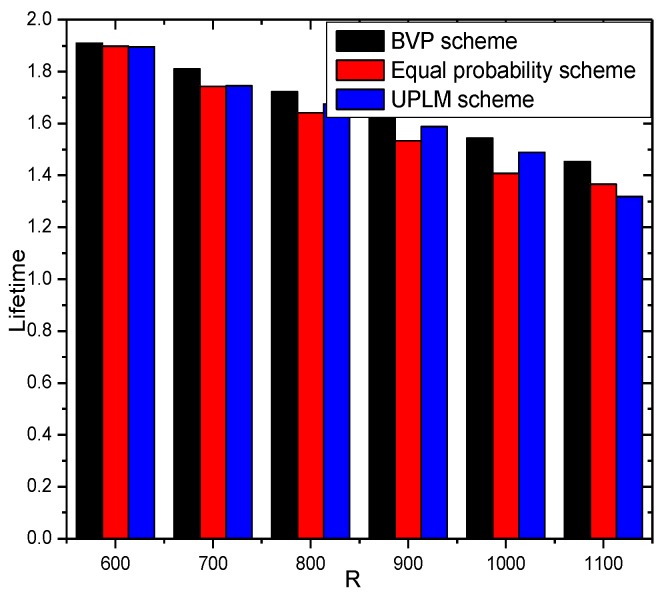
The network lifetime under different network radii and transmission radii.

Figure 16 illustrates the network lifetime under different network radii in different schemes. The network lifetime of the UPLM scheme, BVP scheme, and equal marking probability approach under different network radius do not vary greatly. 

**Figure 17:**
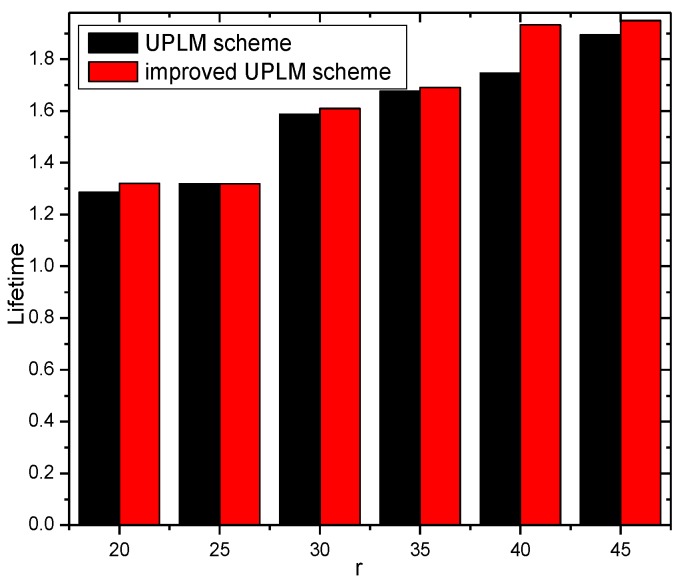
The network lifetime under different transmission radii.

**Figure 18:**
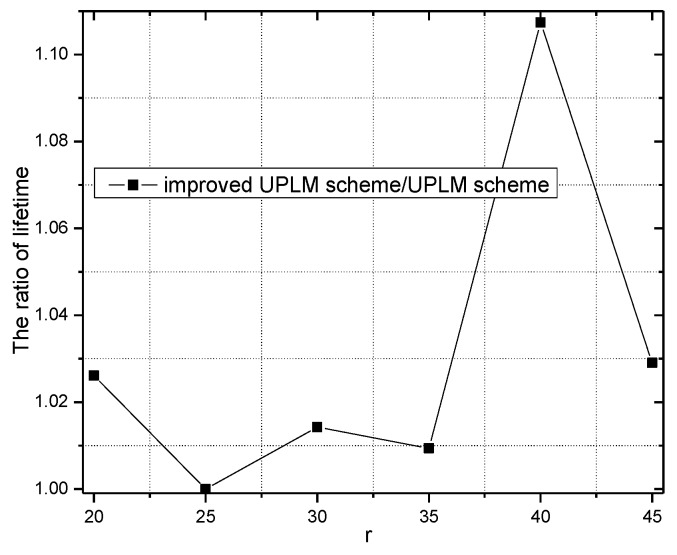
The ratio of the network lifetime under different transmission radii.

Figure 17 and Figure 18 illustrate the network lifetime under different transmission radii r in the UPLM scheme and the improved UPLM scheme. The network lifetime of the improved UPLM scheme is longer than the network lifetime of the UPLM scheme under the different transmission radii r, which implies the better performance of the improved UPLM scheme.

**Figure 19:**
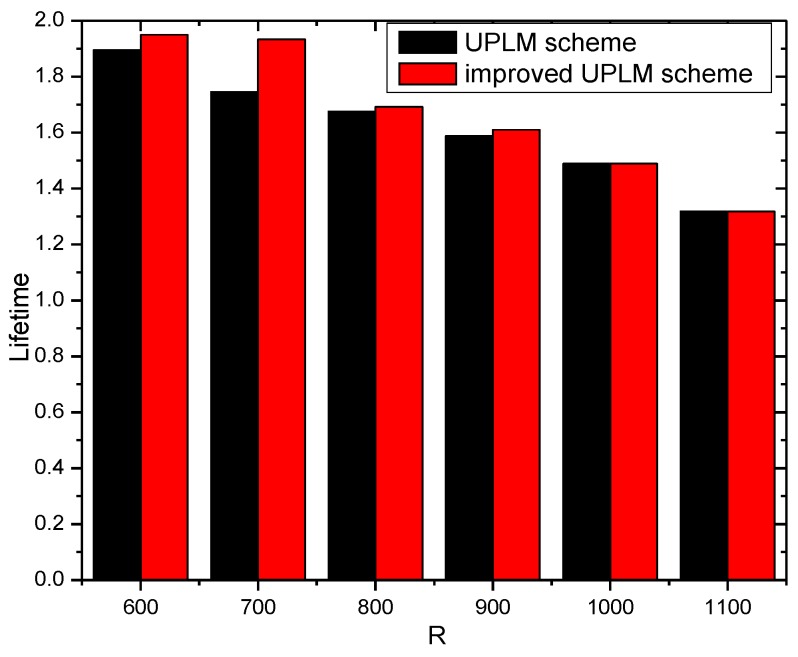
The network lifetime under different network radii.

**Figure 20:**
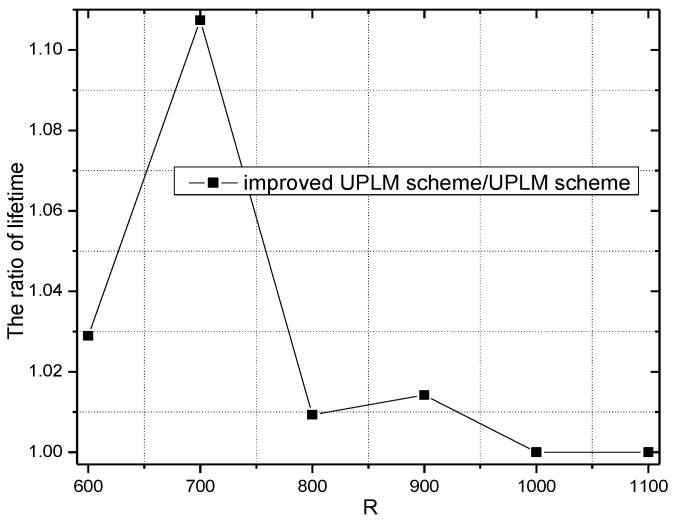
The ratio of the network lifetime under different network radii.

Figure 19 and Figure 20 illustrate the network lifetime under different network radii in the UPLM scheme and the improved UPLM scheme. The network lifetime of the improved UPLM scheme is longer than the network lifetime of the UPLM scheme under different network radii r, which implies a better performance of the improved UPLM scheme.

### 5.3. Analysis of Storage Capacity

**Figure 21:**
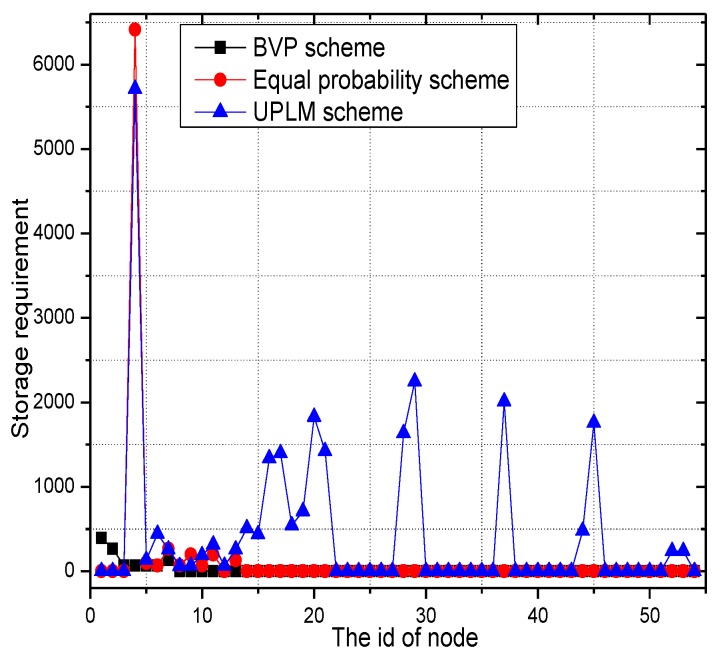
The required storage in different areas.

**Figure 22:**
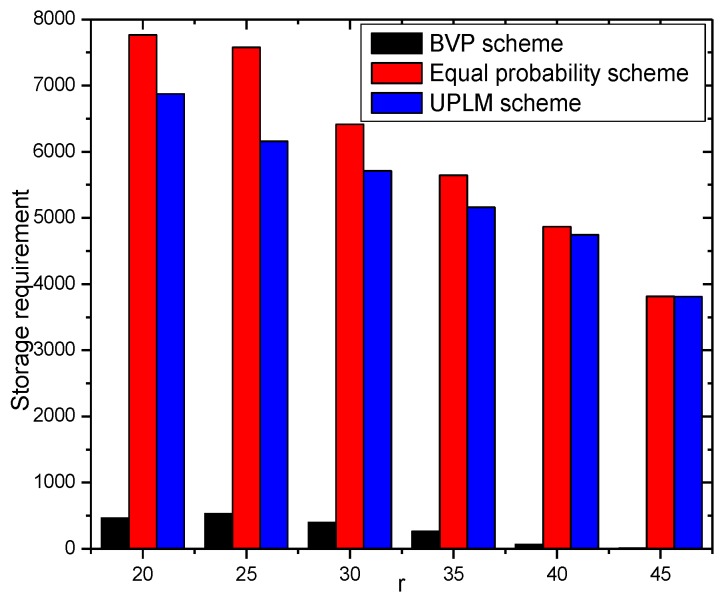
The required storage under different transmission radii.

In this section, the storage capacity required is analyzed. Figure 21 and Figure 22 show that the UPLM scheme is better than the BVP scheme in balancing the storage requirement in WSNs. Despite the truth that the maximum storage requirement of the BVP scheme is not higher than that of the UPLM scheme and the equal marking probability approach, the network lifetime of the BVP scheme is still low as all packets and its marking information are sent to the sink. By comparing the UPLM scheme with the equal marking probability approach, it is obvious to see that the maximum storage requirement of the UPLM scheme is not increased and the balance of the storage capacity of each node in the network is maintained.

**Figure 23:**
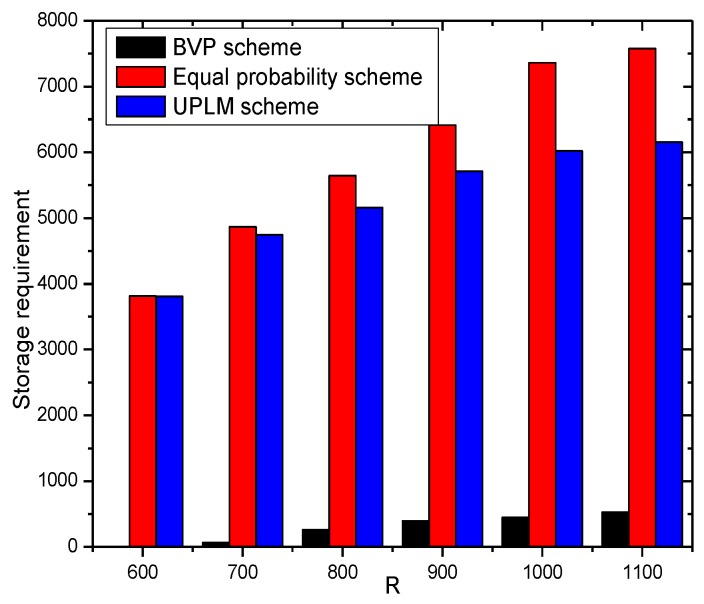
The required storage under different network radii.

Figure 23 shows the storage requirement of each node under different network radii R. The UPLM scheme will not bring extra storage consumption to the network, compared with the equal marking probability.

**Figure 24:**
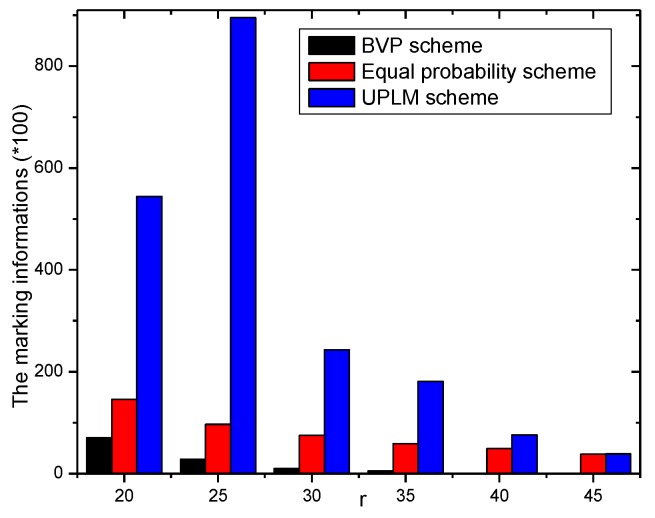
The total utilized storage under different network radii.

**Figure 25:**
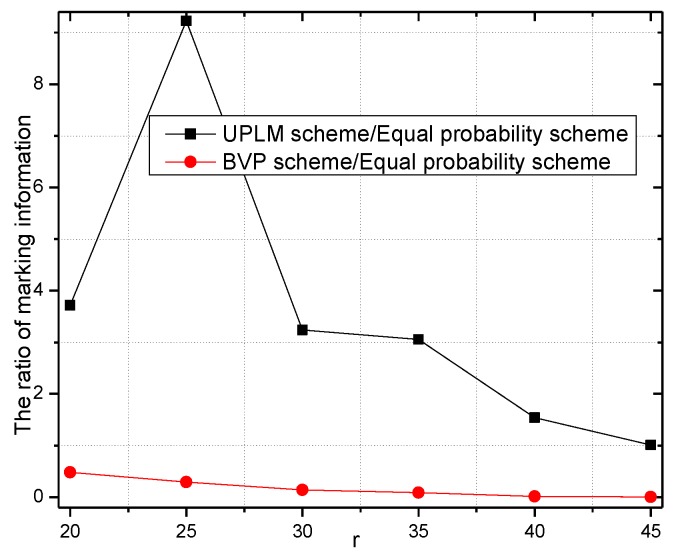
The ratio of utilized storage under different network radii.

Figure 24 and Figure 25 show the storage consumption and its related ratio of the whole network under different transmission radii r. The storage utilization of the UPLM scheme is enhanced compared with the BVP scheme and the equal marking probability approach. The total available storage space is 0.043–0.418 times higher than that of the equal marking probability approach. Although the storage requirement of the UPLM scheme is no more than the equal marking probability approach, the migration strategy in the UPLM scheme will increase the storage consumption of nodes in remote areas. On the other hand, the marking probability of nodes in remote areas is higher in the UPLM scheme. Hence, the process of migration is frequent, which balances the storage capacity of nodes in remote areas.

**Figure 26:**
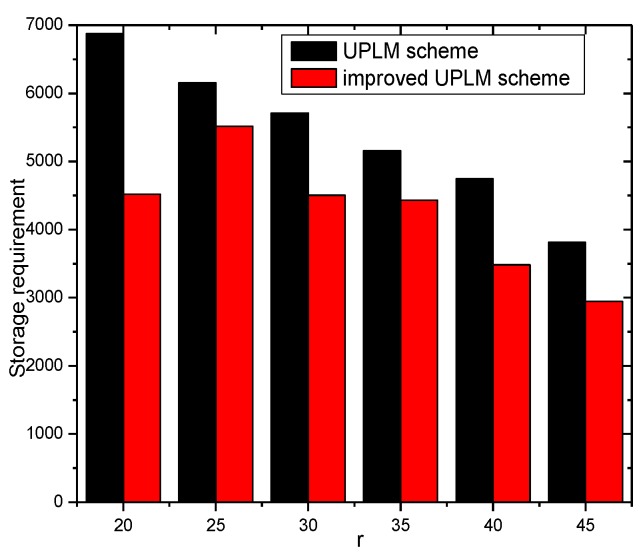
The required storage under different transmission radii.

**Figure 27:**
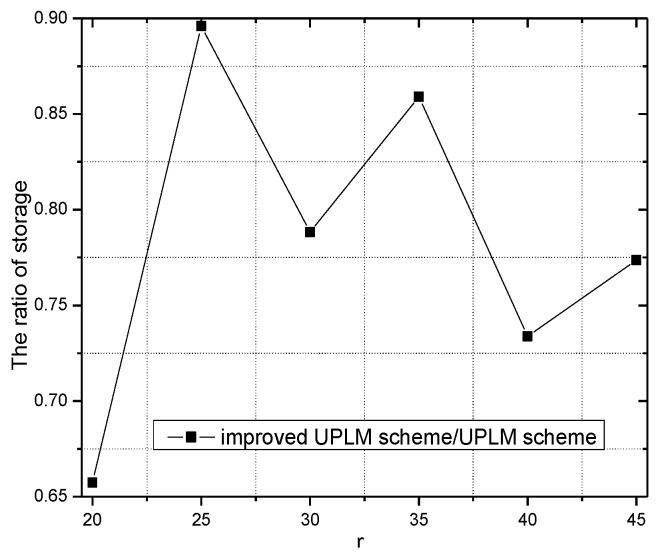
The ratio of required storage under different transmission radii in different schemes.

Figure 26 illustrates the storage requirement of each node under different transmission radii r. Combining Figure 26 with Figure 27, the storage performance of the UPLM scheme and the improved UPLM scheme were analyzed. The storage requirement of the improved UPLM scheme is not greater than that of the UPLM scheme, with no extra storage consumption brought, which shows the superiority of the improved UPLM scheme.

**Figure 28:**
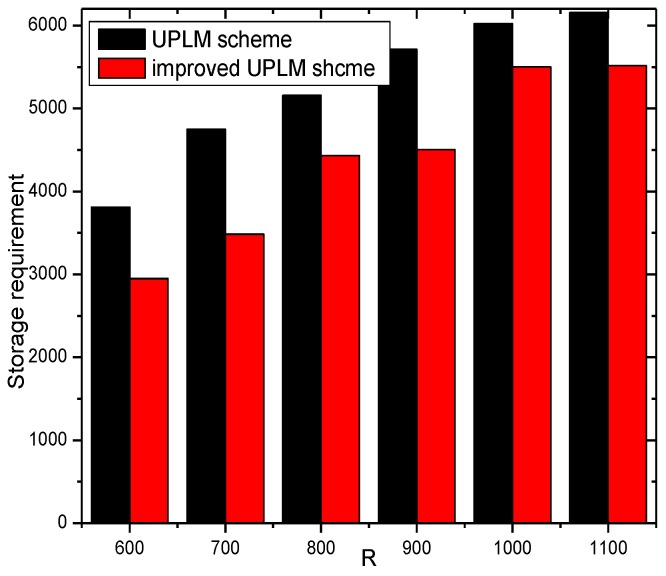
The required storage under different network radii.

**Figure 29:**
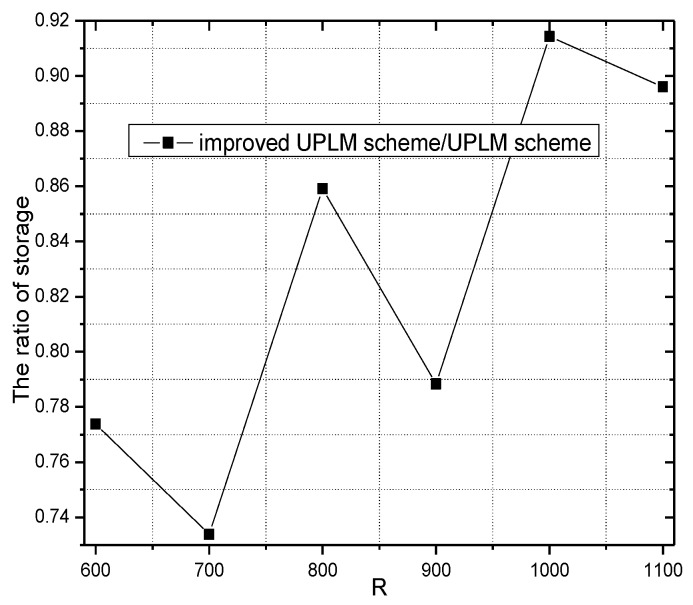
The ratio of required storage under different network radii in different schemes.

Figure 28 shows the storage requirement of each node under different network radii R. Combining Figure 28 with Figure 29, no extra storage consumption was brought by the improved UPLM scheme.

## 6. Conclusions

In this paper, the UPLM scheme was proposed based on the LM scheme. In tree-based WSNs, a large marking probability is assigned to nodes in nearby areas, while the small marking probability is assigned to nodes in remote areas in the LM scheme. This leads to a phenomenon in which a great amount of logged marking information is stored in nearby areas, while the storage of the nodes in remote areas is not utilized effectively. Moreover, the nodes in remote areas still possess too much remaining energy in the LM scheme. The UPLM scheme can significantly increase the total marking information of the network, reduce the required traceback time, and enhance the security performance by increasing the amount of marking information in nearby areas and reducing it in remote areas. The UPLM scheme also migrates the logged marking information stored in nodes with a higher marking probability to further improve the performance of the network. The effectiveness of the UPLM scheme is proven by experiments. The marking information of the UPLM scheme is 1.12–1.28 times more than that of the equal marking probability scheme; the storage capacity of the UPLM scheme is 1.15–1.26 times more than that of the equal marking probability scheme; and the energy utilization is increased by 17.89% in the UPLM scheme. In addition, the buffer area is introduced into an improved version of the UPLM scheme to further enhance the network performance.

## Figures and Tables

**Table 1:** Network parameters.

Parameter	Value
Threshold distance (*d*_0_) (m)	87
Sensing range *r_s_*(m)	15
*E_elec_*(nJ/bit)	50
*e_fs_*(pJ/bit/m^2^)	10
*e_amp_*(pJ/bit/m^4^)	0.0013
Initial energy (J)	0.5
